# Genome-wide chromatin mapping with size resolution reveals a dynamic sub-nucleosomal landscape in *Arabidopsis*

**DOI:** 10.1371/journal.pgen.1006988

**Published:** 2017-09-13

**Authors:** Daniel Antony Pass, Emily Sornay, Angela Marchbank, Margaret R. Crawford, Konrad Paszkiewicz, Nicholas A. Kent, James A. H. Murray

**Affiliations:** 1 Cardiff School of Biosciences, Cardiff University, Cardiff, Wales, United Kingdom; 2 Genome Centre, University of Sussex, Sussex House, Falmer, Brighton, United Kingdom; 3 Geoffrey Pope Building, University of Exeter, Stocker Road, Exeter, United Kingdom; Gregor Mendel Institute of Molecular Plant Biology, AUSTRIA

## Abstract

All eukaryotic genomes are packaged as chromatin, with DNA interlaced with both regularly patterned nucleosomes and sub-nucleosomal-sized protein structures such as mobile and labile transcription factors (TF) and initiation complexes, together forming a dynamic chromatin landscape. Whilst details of nucleosome position in Arabidopsis have been previously analysed, there is less understanding of their relationship to more dynamic sub-nucleosomal particles (subNSPs) defined as protected regions shorter than the ~150bp typical of nucleosomes. The genome-wide profile of these subNSPs has not been previously analysed in plants and this study investigates the relationship of dynamic bound particles with transcriptional control. Here we combine differential micrococcal nuclease (MNase) digestion and a modified paired-end sequencing protocol to reveal the chromatin structure landscape of Arabidopsis cells across a wide particle size range. Linking this data to RNAseq expression analysis provides detailed insight into the relationship of identified DNA-bound particles with transcriptional activity. The use of differential digestion reveals sensitive positions, including a labile -1 nucleosome positioned upstream of the transcription start site (TSS) of active genes. We investigated the response of the chromatin landscape to changes in environmental conditions using light and dark growth, given the large transcriptional changes resulting from this simple alteration. The resulting shifts in the suites of expressed and repressed genes show little correspondence to changes in nucleosome positioning, but led to significant alterations in the profile of subNSPs upstream of TSS both globally and locally. We examined previously mapped positions for the TFs PIF3, PIF4 and CCA1, which regulate light responses, and found that changes in subNSPs co-localized with these binding sites. This small particle structure is detected only under low levels of MNase digestion and is lost on more complete digestion of chromatin to nucleosomes. We conclude that wide-spectrum analysis of the *Arabidopsis* genome by differential MNase digestion allows detection of sensitive features hereto obscured, and the comparisons between genome-wide subNSP profiles reveals dynamic changes in their distribution, particularly at distinct genomic locations (i.e. 5’UTRs). The method here employed allows insight into the complex influence of genetic and extrinsic factors in modifying the sub-nucleosomal landscape in association with transcriptional changes.

## Author summary

DNA is packaged by proteins into chromatin, of which the fundamental unit is a complex of histone proteins that wraps ~150bp of DNA into a nucleosome. Digestion of chromatin with enzymes such as micrococcal nuclease cuts the DNA between the protein particles, and by sequencing the cut sites, they can be mapped across the entire genome. Whilst nucleosomes are the most stable feature, less intensive digestion reveals a wider range of protein particles bound to DNA, in particular small sub-nucleosomal particles located most frequently upstream of genes. Here we show that these techniques can be used in the plant Arabidopsis to map the chromatin landscape of the range of particles sizes across the genome. We show for the first time in a multicellular higher eukaryote that this chromatin landscape is dynamic in response to environmental changes, in this case light or dark growth. Whereas the nucleosome positioning does not change significantly, we show profound changes in the smaller more labile factors under these different conditions. These changes in many cases correspond to the known binding sites of transcription factors that regulate genes in response to light, leading us to propose that full-spectrum chromatin landscape analysis can reveal directly the changes in transcription factor complex binding across the genome.

## Introduction

Within the nucleus, higher eukaryotic genomes are packaged in the form of chromatin in which the structural unit is a nucleosome, a ~150 bp of double-stranded DNA wound around an octameric histone protein core. Nucleosomes are arranged in a repetitive manner spaced by a linker region of DNA with a length of ~30 bp, varying by species and genomic location [1,2]. The positioning of nucleosomes relative to DNA sequence is a dynamic process, regulating access to the DNA sequence of various factors and is essential for proper functional biological processes such as transcription, replication, DNA repair and recombination [3]. Moreover, both post-translational modifications of nucleosomal histones and positioning of nucleosomes on the DNA sequence contribute to epigenetic genomic information [4].

Technologies combining digestion of unbound double-stranded DNA using enzymes such as DNAse I or micrococcal nuclease (MNase), and next-generation sequencing (NGS) of the remaining protected material allow the generation of genome-wide maps of nucleosome positions. These studies have previously been carried out in a wide range of eukaryotic organisms such as *Saccharomyces cerevisiae* [5], *Caenorhabditis elegans* [6,7], *Drosophila melanogaster* [8], *Zea mays* [9], and also in human cell culture systems [7,10]. Chodavarapu et al. (2010)[11] presented the first nucleosome-positioning map of mononucleosomes in *Arabidopsis thaliana*. They showed that (1) exons are nucleosome-enriched, (2) the intron-exon boundaries are demarcated by strongly positioned flanking nucleosomes, and (3) nucleosomal DNA is methylation-enriched. However their findings focus more on genome region rather than the underlying DNA sequence. Similarly it was shown that genome-wide, nucleosome patterning is uniform in protein-coding genes but not in pseudognes, transposable element genes and transposable elements [12,13]. Nucleosome patterning is uniform in euchromatin, whereas pericentromeric heterochromatin shows nucleosome enrichment [11]. Additionally, the periodic distribution of nucleosomes is independent of the level of expression of the gene, but the unoccupied distance between nucleosome positions is higher in transcribed genes [12].

Typically in these studies, digestion of chromatin was performed to completion with optional gel purification steps utilised to enrich for mono-nucleosomal DNA fragments. This gives a limited and static picture of the chromatin landscape focused on the positioning of stable nucleosomes. However, the dynamic structure of chromatin is required for changes in gene expression [14]. Partial digestion of chromatin in yeast and flies reveals variability in nucleosome occupancy profiles explained by the presence of MNase-hypersensitive nucleosomal DNA regions [15–17]. The existence of both hypersensitive and hyper-resistant nucleosomes was shown in the crop plant *Zea mays* [18]. These regions are found in the non-coding DNA around active genes and are colocalised with regulatory elements found in converse noncoding sequences, sequences such as KNOTTED 1 transcription factor binding site [19–21]. Moreover, MNase sensitive regions also correlate with recombination hotspots and hypomethylation [21]. These studies highlight the complex nature of labile DNA-bound proteins.

In addition to the visualisation of nucleosome dynamics, partial digestion approaches allow size-resolution of nuclease protected chromatin particles using modified paired-end mode sequencing protocols to reveal the positions of sub-nucleosome sized particles (subNSPs). These subNSPs protect DNA fragments of a size smaller than 120bp and may represent non-nucleosomal chromatin-associated proteins including DNA replication machinery such as DNA polymerase Origin Recognition complex (ORC) [20] or the transcriptional machinery such as sequence-specific TFs and complexes containing TFs [22]. Further examples include chromatin remodeler complexes [23], and RNA polymerase or proteins involved in chromatin structure [24,25] in DNA recombination [26] or DNA repair [27].

Here we combine partial differential MNase digestion and size-resolved chromatin-seq with transcript profiling from the same samples in *Arabidopsis thaliana* for the first time. Low levels of MNase treatment reveals complexity regarding small particle recruitment and the lability of bound factors which has only previously been suggested in yeast [16] and human sperm [28] through comparisons of mutant types. Here we examine for the first time the dynamics of the overall chromatin landscape in a single cell type to changes in environmental conditions correlated to RNA-seq data, giving insight into the dynamic relationship between chromatin and the changing transcriptome.

## Results & discussion

### Developing a comprehensive chromatin map of *Arabidopsis thaliana*

Eight replicate *Arabidopsis thaliana* Col-0 cell cultures were sampled after 16-hour passage (see Methods) (4x Light grown, 4x Dark grown) and subjected to a “higher” or “lower” level of chromatin digestion with MNase followed by Illumina Paired-End sequencing modified to take a wide DNA fragment size range. This allowed us to perform chromatin particle spectrum analysis (CPSA) as previously described in yeast [29] and enabled determination of regions of genomic DNA protected by nucleosomal, multi-nucleosomal, and sub-nucleosomal sized particles (NSP, multiNSP, subNSP) by retaining size information when mapping to genomic DNA.

**Fig 1:**
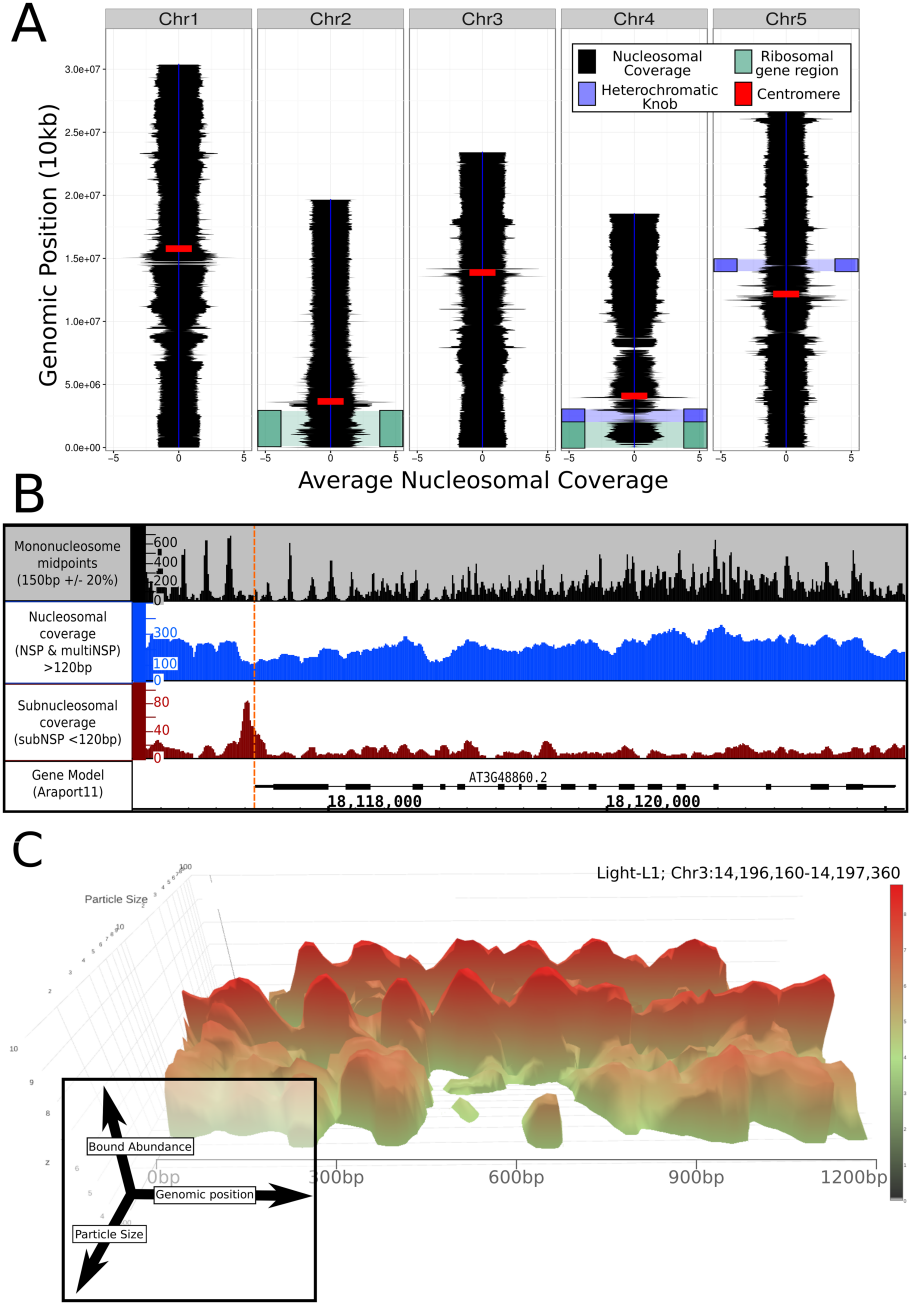
Genomic organisation of chromatin in *Arabidopsis thaliana*. (A) *A*. *thaliana* genome wide chromatin mapping of nucleosome structure (Mono- and multiNSP binding at 10kb genome regions) with approximate positions of notable genome features [31] demonstrates predominantly homogeneous coverage but with distinct areas of heterogeneous mapping. (B) Gene view demonstrates at organisation of mononucleosomes through mapping of midpoints (black), total mono- and multiNSP genomic protection (blue), and notably, recruitment of a subNSP (<120bp) at the TSS co-ordinates (red). (C) Genomic protective particles demonstrated continuous size variability (depth-axis) with distinct structure observable at the mono-nucleosomal size, highlighting the complex profile of DNA-bound protein.

Spatial mapping of all protected DNA sequences demonstrated a homogeneous coverage of the *A*. *thaliana* genome with nucleosome sized bound particles, albeit with some regions of lesser chromatin coverage (Fig 1A). Regions such as the centromeres, the heterochromatic knob on chromosome 4 [30], and the rDNA encoding regions demonstrate abnormal structures likely due to their highly repetitive state inhibiting accurate mapping [31]. Mapping of the midpoints of all 150bp (+/-15bp) fragments across the genome (Fig 1B: black track) provides an NSP pattern remarkably consistent with previous results [11,12,32]. However, the approach of mapping according to size provided novel insight into the range of DNA-bound material, as utilising low intensity MNase digestion revealed not only NSPs, but also multiNSP regions resistant to MNase. Coverage of the underlying genome by nucleosomal (~150bp +/- 30bp) or multi-nucleosomal (N * (150bp + linker)) chromatin demonstrated a regularly occurring structure throughout the genome, with higher association to genic regions (described further below). Interpolation between sequenced paired reads and cumulative measurement of all mapped fragments equal to or larger than nucleosomal and multi-nucleosomal sized particles generated a single nucleotide resolution map of the total coverage of the underlying DNA at genome scale (Fig 1B: blue track). Furthermore, mapping by particle size in addition to genomic location demonstrated the continuous range of bound particle sizes apparent at this low digestion, as is observed directly in the 1.2kb exemplar region in Fig 1C. This 3D plot shows the non-discrete range of fragment sizes and abundances, revealing the absence of subnucleosomal fragments mapping to its centre, periodic mono-nucleosomes and a range of multi-nucleosomal protected regions.

The differential MNase digestion method was previously implemented in yeast and revealed DNA fragments protected by subNSPs such as TFs [16,29]. To profile the subNSPs across the genome, we computationally subsampled all sequenced material where paired-end reads were less than 120bp i.e. sub-nucleosomal sized regions protected from MNase cutting activity. This revealed that subNSP binding factors have distinct organisation to key positions throughout the genome, specifically positions immediately upstream of, or coinciding with 5’ untranslated region (UTR) sites, as observed in the example of Fig 1B (red track). This is consistent with the profiles expected by the binding of TFs and promoter complexes directly upstream of TSSs as observed in yeast [16,29].

**Fig 2:**
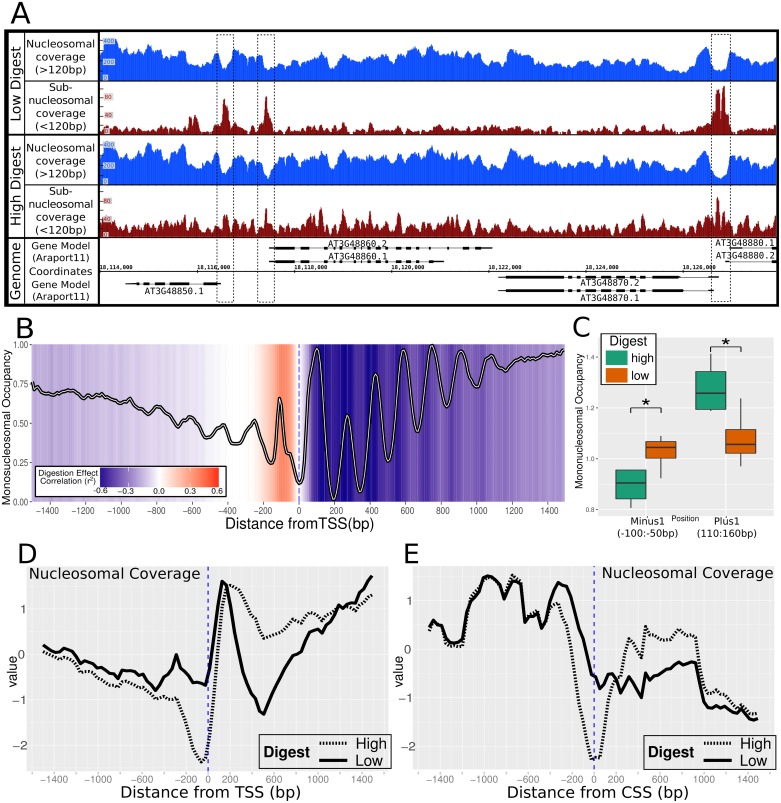
The effect of differential MNase digestion on nucleosomal organisation. (A) NSP and multiNSP coverage occurs throughout the genome but indicates open chromatin at positions of TF recruitment i.e. 5’UTR TSS locations (Blue). Corresponding subNSP recruitment at these locations was markedly less prominent with high-MNase digest preparation (Red). (B) Periodic NSP organisation was conserved at TSS sites (samples = 8, genes = 21,314), with strongly positioned nucleosomes directly downstream from the TSS. Colour scale represents correlation between digestion level estimate and nucleosomal occupancy at that position, where increased digestion correlates with greater monoNSP abundance. (C) Comparison of average occupancy between digestion treatments at 50bp regions in the ‘minus-1’ and ‘plus-1’ nucleosome region with significance (* = P<0.05, t.test). Total nucleosomal and multi-nucleosomal coverage surrounding (D) TSS and E) CSS demonstrates the sensitive labile nucleosomes positioned at the TSS and CSS which are unobserved under high digest.

Comparison of the otherwise equivalent but differentially digested *A*. *thaliana* samples between ‘high’ and ‘low’ level of MNase treatment demonstrated the effect of digestion extent on the DNA-bound material. At a genomic level, overt variation was not observed in NSP or multiNSP counts between differentially digested samples, consistent with view of chromatin as an essential stabilizer of the genome [5,33] (S1 Fig). However, the marked variation in number of subNSP identified between digestion levels indicates the sensitive nature of these labile factors and suggests that their binding is more readily displaced from the underlying genome than nucleosomes (S1 Fig). Under high digest, subNSPs prominent in low digest samples appear to have a reduced detection indicating greater susceptibility of the underlying DNA to nuclease-mediated degradation due to weaker protection from the bound protein in comparison to nucleosomes. Subsequently, high digest resulted in subNSPs frequently indistinguishable from the likely noise of surrounding low-level small-particle structure, particularly at TSSs (Fig 2A—compare red tracks).

Differential digestion levels in *Zea mays* has previously enabled description of hyper-sensitive sites in the genome where fragile nucleosomes are found [19]. We here observe a similar fragile nucleosome throughout the genome at the ‘-1’ position directly upstream of the TSS (Fig 2B & 2C). Our comparative analysis was consistent with this outcome, which however was not previously reported in *A*. *thaliana*, likely due to high levels of MNase digestion [12]. For this reason, we believe that the low-digestion level and wider particle size analysis implemented here is essential in producing a holistic view of the nucleosomal landscape, and a facet often omitted from other genome-accessibility studies.

### Limited digestion reveals varied binding structures in the chromatin landscape of *Arabidopsis* through identification of sub-nucleosomal sized bound particles

The positioning of nucleosomes was observed to be most conserved at the TSS in individual gene examples (Fig 1B). This periodicity is less defined downstream, with the less stringent chromatin organisation being consistent with previous results [12]. Plotting the average structure surrounding the TSS of all *A*. *thaliana* genes (with consistent expressed isoforms between samples, N = 21,314), we observed periodical ~150bp mono-nucleosomal organisation patterning as previously described in *Arabidopsis* (Fig 2B) [11] and typical of other eukaryotic organisms [6,7,17,29]. However, nucleosome positioning in the average profile surrounding the TSS also incorporates the variation between gene lengths and intron-exon structure when surmising multiple gene regions, which contributes to high genic average coverage but lower definition of nucleosomal periodicity. We note the comparative low height of the +2 nucleosome peak which is a consistent feature of our analyses, but currently unexplained.

Consideration of MNase digestion level on mono-nucleosomal organisation shows higher digestion directly correlates with increased mono-nucleosomal structure in the genic region downstream of the TSS. Fig 2B indicates the areas where increased digestion correlates with strengthened or weakened monoNSP positions surmised through the genome, where the colorimetric background shows the strength of correlation between digestion level and NSP abundance per 10bp region. We observe a particularly sensitive nucleosome (negatively correlated to digestion) directly upstream of the TSS which has significantly lower occupancy in the high digested samples (Fig 2C). This is in concordance with the hypersensitive minus-1 nucleosome identified in maize [18]. Additionally, the partially resistant multiNSP and sensitive subNSP positions are less represented under high digest conditions. For nucleosomes this is presumably due to cleavage of di- or tri-nucleosomes to monomers, however the comparative susceptibility of subNSPs results from greater access of MNase to digest under higher levels due to weaker binding or lower levels of DNA protection. The intron-exon boundary demonstrated a strong defining role for nucleosomal positioning as previously described [10], but is not as strong a factor as that by which nucleosomal structure is organised at the TSS (S2 Fig).

Observing the nucleosomal organisation at the TSS regions of individual genes supports variable access of subNSPs due to widening of the open chromatin region between nucleosomes. This pattern is replicated at genomic scale, where open chromatin regions appear at the TSS and Coding Start Sites (CSS) (Fig 2D & 2E), suggesting access for TF recruitment. The differential occupancy signals found at these open regions supports that differential digestion is required to demonstrate the exclusion of the labile and sensitive factors in these regions.

### Genome-wide effects of differential growth conditions on chromatin and subNSP structure

The irradiance change in environmental conditions of the diurnal cycle is central to the cyclic expression changes in the plant transcriptome and the normal phenotype [34]. The changing accessibility of key genes has an impact on this expression. Nucleosome spacing at specific genes was correlated with their expression level in targeted analysis [12]. We here addressed the light-responsive gene networks to further understand the role that chromatin has on the activation and inactivation of genes. In addition to NSP binding, we analysed subNSP binding to get a more thorough global view of chromatin dynamics.

**Fig 3:**
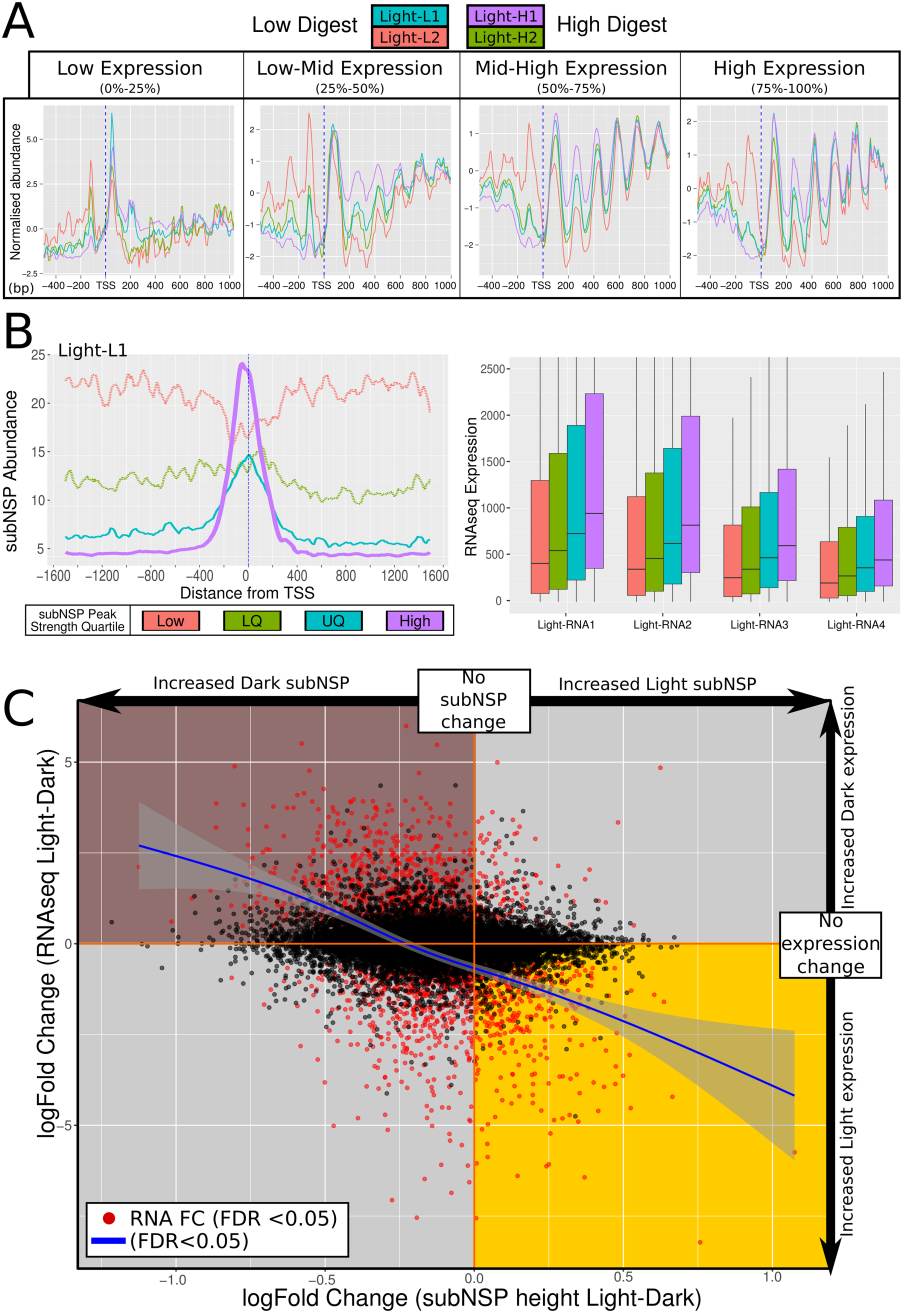
Varying gene expression corresponds to genomic chromatin organisation changes and variation in response to irradiance. (A) NSP structure surrounding TSS separated by quartile of gene expression demonstrated reduced structure correlating with lower expressed genes (light grown samples under high and low digest represented). Inactive and extremely low-expressed genes notably reveal a large +1 nucleosome likely active in inhibiting gene expression. (B) Genes were quartile separated by abundance of subNSP recruitment at TSS as represented in average profile of quartiles (Light-L1 example: upper). Level of gene expression grouped by quartile demonstrated increasing recruitment and correlated with increased expression within each light-grown sample. (C) Comparison of Light and Dark grown samples for fold change of subNSP abundance was performed against corresponding gene expression. Genes under significant expression change (FDR<0.05, red) demonstrated greater recruitment of subNSP in accordance with higher expression (Trendline: leoss smoothed, 0.95 CI: grey boundary).

Chromatin is understood to be more rigorously structured in exonic regions of genes than intergenic and intronic [11,12,32]. Utilising RNAseq data derived from the same samples used for chromatin-seq, the genome was divided into quartiles based upon gene expression level and the NSP positioning was plotted four bio-replicates of light grown cell cultures subjected to low and heavy digest (see Material and methods). Genome-wide, genes under median to high expression showed a strongly focused positioning of NSPs surrounding the TSS (Fig 3A, 50–75%, 75–100%). This structure was less pronounced in low-expressed genes (Fig 3A, 25–50%) and a solitary strongly positioned nucleosome at the point of TSS initiation (up to 200bp downstream) was typical in the lowest quartile of expressed genes (Fig 3A, 0–25%). This loss of mononucleosomal-sized patterning supports the assertion that typical control of gene expression requires well-regulated chromatin [12]. Both the immobile NSP at the TSS of inactive genes, and a strongly defined TSS+1 NSP have been previously reported [12], which was showed to inhibit the binding of transcription complexes and other factors, and to inhibit RNA Pol II activity [35]. Consideration was given to lateral movement of the TSS+1 nucleosome in response to changes in expression, allowing for increased upstream access, however we did not observe convincing evidence for this (S3 Fig).

We explored the position of subNSPs for the same range of light grown samples. SubNSP binding was enriched at TSS regions throughout the genome. Genes were separated into quartiles by degree of kurtosis of the subNSP peak at the TSS, showing that stronger recruitment correlates with higher levels of expression across the genome (Fig 3B—upper: example peak structure separation, lower: 4 samples with RNAseq separated by subNSP recruitment). The increased expression in the higher quartiles of subNSP recruitment could either be explained by more frequent binding within the cell population at that position, or stronger binding to DNA by the subNSP, or more defined MNase-hypersensitive linkers at the TSS.

### Differential growth conditions effect subNSP changes at single gene resolution

Having establishing the nucleosomal and sub-nucleosomal structural changes within single samples, we contrasted the chromatin profiles between isolates grown in different irradiance conditions. We did not identify a statistically significant change in nucleosomal patterning genome-wide between samples grown in light or in dark (following two weeks of continuous culture without light). We therefore conclude that the NSP structure is not significantly changed in response to this extrinsic environmental change, even when applied for an extended period. Consequently, we investigated the binding of subNSPs to discern any effect of the environmental changes to the DNA-bound landscape. The correlation between changing gene expression (RNAseq, log_2_-fold change) and the recruitment of subNSP (TSS subNSP abundance, log_2_-fold change) was assessed for each shared-isoform gene (Fig 3C). We identified that genes under significantly increased expression in either condition (p>0.05, FDR) correlated with stronger recruitment of subNSP binding at the TSS consistent with DNA-binding complexes such as TF being present at the time of sample harvest.

**Fig 4:**
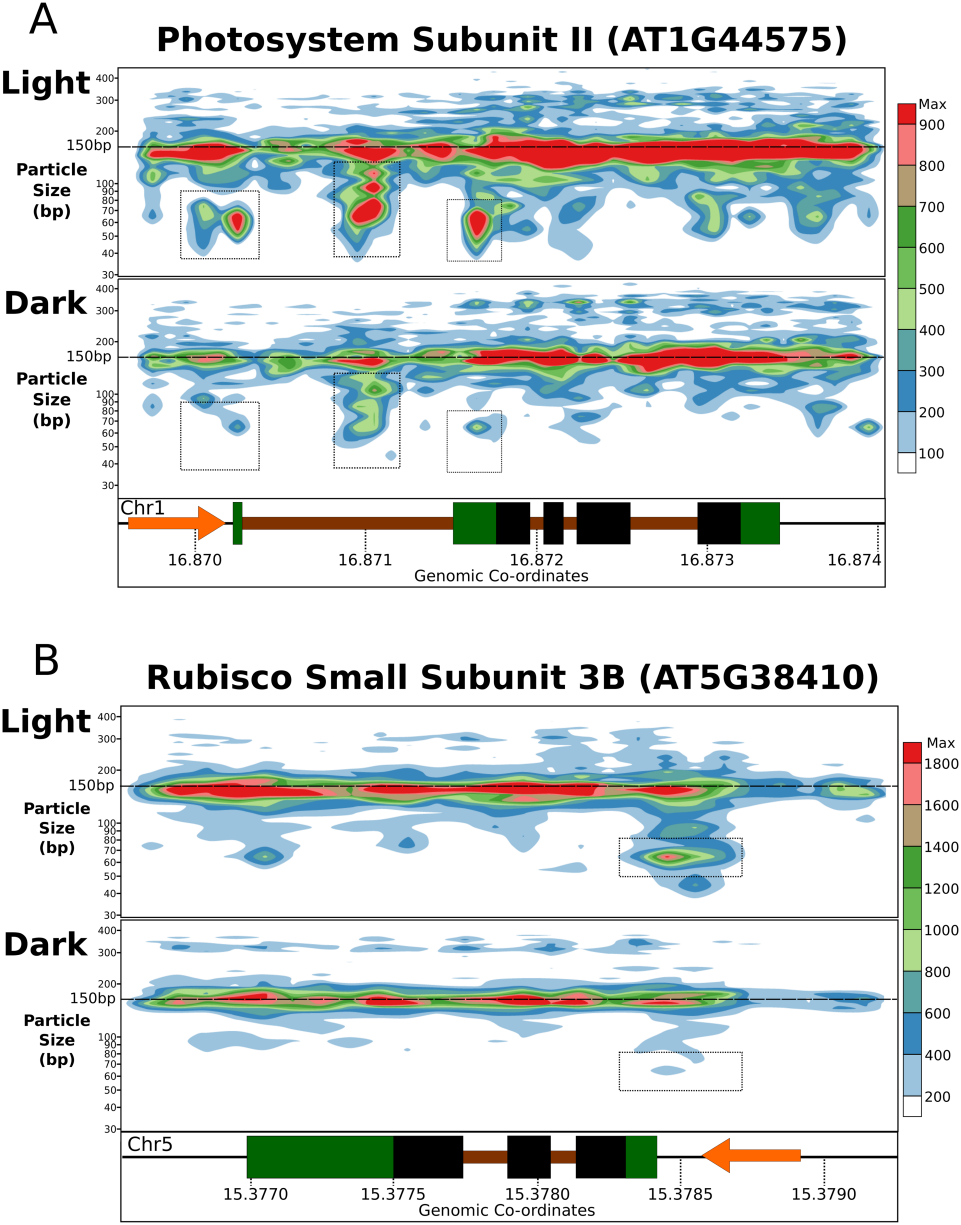
SubNSP recruitment occurs preferentially at active genes. Total coverage mapping of all sized DNA-binding particles for low-digested samples (Light-L1/L2 and Dark-L1/L2) demonstrates recruitment of subNSPs to specific TSS and UTR positions in the key light-response genes (A) Photosystem Subunit II (NPQ4, AT1G44575) and (B) Rubisco Small Subunit 3B (RBCS3B, AT5G38410). Observed subNSP recruitment is absent from dark-grown samples which are almost totally inactive (PSII—FC: -10.8, FDR = 2.59E-136, RBCS3B –FC: -9.46, FDR = 3.01E-076). Colour scale represents abundance of mapped fragments normalised for sequencing depth.

Hence, whilst no change in gross NSP pattern was seen, changes of expression caused by applied environmental stimuli were correlated with a genome-wide changes at the level of subNSP binding. We therefore sought to understand whether the average landscape observed across the genome was representative of the local profile at well-regulated genes. Within genes of relevance to the light response, we assessed key genes involved in photosynthesis or photosensing that showed strong expression in the RNA-Seq data. For instance, *Photosystem II Subunit S* (*CP22*, AT1G44575, fold change: -10.8, FDR = 2.59E-136) showed that subNSP recruitment was observed around the TSS and the UTR region directly 5’ to the CSS, but was not identified in samples grown in dark (Fig 4A, dashed boxes). *CP22* encodes a photoprotective pigment-binding protein associated with the photosystem II in the grana thylakoids [36]. To prevent the formation of reactive species and the damage to membranes, a quenching of excited chlorophyll occurs by transferring the energy to carotenoid pigments [37]. Another example is the disappearance of a 5’UTR associated subNSP in dark compared to light for the *Rubisco Small Subunit 3B* (AT5G38410, fold change: -9.46, FDR = 3.01E-076, Fig 4B), encoding a subunit of the key Calvin cycle enzyme ribulose 1,5-bisphophate carboxylase-oxygenase (RuBisCO) [38]. Again, the presence of subNSP at the 5’UTR appears to coincide reliably with the increased expression.

**Fig 5:**
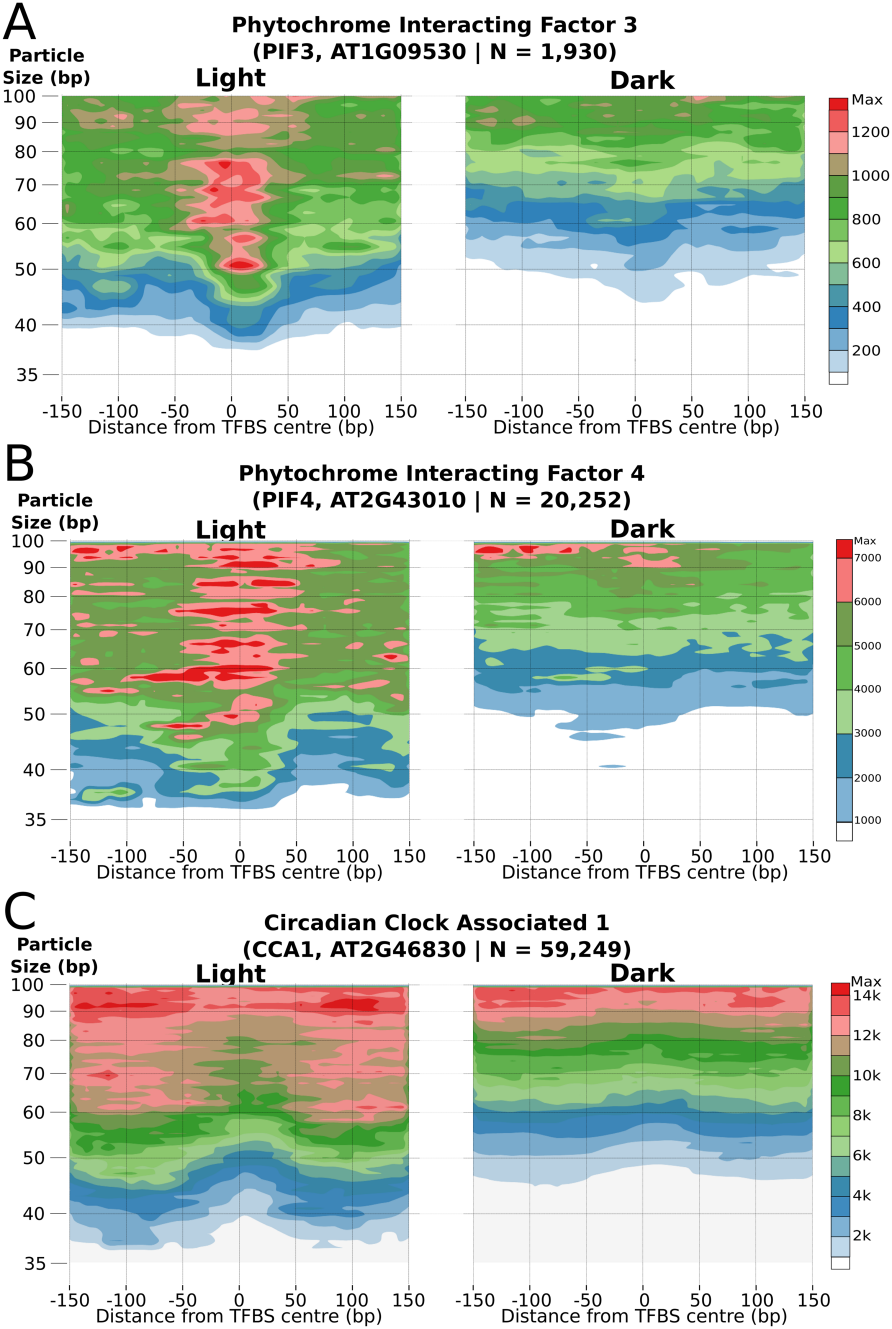
SubNSP organisation at transcription factor binding sites. TFBS positions were assayed for subNSP binding for light-responsive Transcription Factors (A) PIF3 (n = 1,930), (B) PIF4 (n = 20,252) and (C) the CCA1 (N = 59,249). Direct visualisation highlights the differential binding of the subNSP across the genome between growth conditions and identifying the TF response to the extrinsic irradiance changes.

We then examined whether these changes in subNSPs could be related to known positions of TF binding to specific genomic locations. Previously identified TF Binding Sites (TFBS) were sourced from Athamap [39,40] (S4 Fig) or O’Malley et. al (2016) [41] to assess subNSP binding to their corresponding genomic targets. *PHYTOCHROME INTERACTING FACTOR 3* (PIF3) is a helix-loop-helix TF interacting with photoreceptors phyA and phyB as part of the plant light response [42]. Analysis of the PIF3 target binding motif across the genome demonstrated significantly more occupancy in light conditions (Fig 5A, Number of sites = 1,930). This was despite a non-significant change (log2FC: -0.25, FDR = 0.7) in expression of the PIF3 gene, consistent with post-translational regulation of the TF through phosphorylation [43]. The related PIF4 TF was however associated with a significant downregulation in dark-grown cells (AT2G43010, log2FC: -1.4, FDR = 0.079) and revealed a similar subNSP binding structure at identified sites (Fig 5B, N = 20,252)[44].

In contrast to the light responsive PIF TFs, the Myb-type *CIRCADIAN CLOCK ASSOCIATED* (CCA1, AT2G46830) TF similarly demonstrated increased binding at known TFBS [45] in light-grown samples, but presents an alternative saddle-shaped binding pattern. This heightened accumulated around the binding site indicates the motif region having susceptibility to MNase digestion and the subNSP being offset in dark conditions (Fig 5C, N = 59,249). TF encoding genes with higher expression in dark grown samples (ERF1, WRKY59, AT4G18450) did not demonstrate an increased abundance of subNSP binding and patterning was retained, although some variability can be observed due to variation in digestion level (S5 Fig).

While the majority of subNSP binding positions are associated with genic promotor regions, approximately 7.91% of positions identified were greater than 10kb from an annotated coding sequence and 1.18% greater than 100kb distant. These sites can clearly be observed as protected DNA fragments of unknown function, which supports the role of the subNSP/TF detection approach as a new methodology to reveal not only the potential binding of transcription factors but also indicating other regions of interest for further analysis. However, while these positions may function as binding locations for long distance promotor elements, there are also potential roles unrelated to transcription factor binding which must be considered including the spatial organisation of DNA, and underlying physical structure of DNA which make regions resistant to MNase digestion i.e. secondary structures. Furthermore, investigation into associations with underlying genomic factors and histone modification statuses will be of significant future interest due to the connection of modified histones to transcriptional control [46]. One aspect to be assessed under matched conditions is the connecting the exclusion of H2A.Z and methylation of DNA in actively transcribed DNA [47], and positions found to be enriched for subNSP binding ie. the 5’UTR and Transcription Start Sites. As ChIP-seq approaches to mapping TFs in *A*. *thaliana* advance, we should come to learn the identities of the factors we have observed protecting DNA and their role in the genomic landscape.

## Materials & methods

### Condition for cultivation of *Arabidopsis thaliana* cell line

Dispersed cell suspension cultures, (prepared from *Arabidopsis thaliana* leaves Columbia ecotype, Col-0 [48,49] and were a kind gif of Dr. Linda Hanley-Bowdoin obtained from North Carolina State University, USA) were used. The cultures are a homogeneous population of physiologically and morphologically identical cells [50,51]. The cultures were maintained in 250 mL Erlenmeyer flasks, filled with 50 mL of cell culture medium (Gamborg’s B5 basal medium with minor organic (Sigma G5893) in 1.1 mg/L 2,4-D, 3 mM MES and 3% sucrose) as previously described [49,52]. Cells were grown on a rotary shaker 160 rpm at 23°C (LS-X (Lab Shaker), Kuhner Shaker X). Constant light was used whereas dark grown cells were incubated under the same shaking and temperature condition but the flask were covered of aluminium foil. Cell line was subcultured every 7 days with a 2:50 (inoculum: fresh medium) dilution ratio. Cultures were carried out in duplicates.

### Cell sampling

Light grown cells were sampled 5 days or 16 hours after subculture. Dark grown cells were adapted to dark conditions for 2 weeks with a 7-day subculture period. Dark grown cells were sampled 16-hour after the 2nd subculture. Cells were sampled by harvesting 30mL of cell culture and washed with autoclaved ultrapure water. Cells were frozen in liquid nitrogen and ground into a fine powder. The cell powder was either stored at -80°C or used directly for MNase digestion and RNA isolation.

### Chromatin digestion

Pelleted cell culture was ground in a liquid nitrogen-cooled pestle and mortar to generate a cell powder. 1mL of the powder was suspended in a 0.5mL modified spheroplast digestion buffer & Nonidet P40 (SDBN: 1M sorbitol, 10mM NaCl, 50 mM TrisŸHCl pH7.5, 5mM MgCl2, 1 mM CaCl2, 1 mM 2-mercaptoethanol, 0.5 mM spermidine, 0.075% Nonidet P40). 300 μl of cells were transferred into 1.5mL microcentrifuge tube containing 30 or 120 units of MNase (Affymetrix). Cells were digested with MNase at 37°C for 3 min. The MNase digestion reactions were stopped by addition of and thorough mixing 30 μl of STOP solution containing 5% SDS and 250 μl EDTA. DNA was extracted by addition of an equal volume of 2X CTAB supplemented with PVP (200 mM Tris-HCl, 40 mM EDTA, pH 8.0; 2.8 M NaCl, 4% w/v CTAB, 2% PVP-40 CAS number 9003-39-8) was added to the digestion reaction and incubated at 45°C for 15 mins. DNA was separated from protein by two phenol: chloroform (1:1; 600μl total) steps. DNA was precipitated with sodium acetate and propan-2-ol, washed in 80% ethanol and dried. Samples were incubated with 10X RNase A at 37°C for 1h with 100U unmodified T4 polynucleotide kinase (NEB) for further 30 min at 37°C.

MNase digested DNA was separated on 1.5% agarose gel, stained with ethidium bromide for 10 min at 80V, DNA fragments ranging from 20 bp to 1 kb were excised and gel pieces containing DNA were placed in a Costar Spin-X 0.45 μm cellulose acetate centrifuge tube filter (Sigma CLS8136). Two series of freeze-thaws (-80C for 10 mins/RT for 10 mins) were performed to macerate the gel. Filter tubes were centrifuged twice (14kg; 5 min; RT) with a 180 degree rotation of the tubes between the two spins. DNA in the aqueous phase was then extracted with 400μl phenol: chloroform (1:1), and precipitated at -80°C for 30 mins with sodium acetate to propan-2-ol and finally washed with 80% ethanol before being resuspended in ultrapure milliQ water.

### RNA isolation

Total RNA was extracted using the RNeasy Plant mini Kit (Qiagen, http://www.qiagen.com).

### Nucleic acid quantification and quality check

DNA samples were quantified on QubitTM 2.0 Fluorometer with dsDNA BR assay and RNA with Qubit RNA BR assay according to manufacturer’s instructions. RNA quality was checked on 1.5% agarose gel.

### Library preparation and sequencing

Exeter Sequencing Service and Computational core facilities at the University of Exeter performed the Genomic and RNA library preparation, quantification and 50bp paired end sequencing on HiSeq 2500. Additional information can be found on the Exeter Sequencing Service website (http://sequencing.exeter.ac.uk/). A total of ~861M paired end reads were obtained from the MNase-seq genomic library and ~173M for the RNAseq library (S6 Fig).

### Data processing and bioinformatic analysis

Genomic mapping was performed with bowtie allowing for 3 nucleotide errors [53], resulting in 85.09% alignment to the TAIR10 reference Columbia-0 genome [54]. Aligned BAM files were converted to wig trace files for monoNSP midpoints (150bp +/- 10%) using bespoke perl scripts. DNA coverage was produced using bamToBed and coverageBed from the bedtools package [55]. Datasets were either complete (total) or where specified split into <120bp, and >120bp subsamples, and converted to wig traces (S7 Fig). Gene feature alignments were produced using danpos [56] and the ARAPORT11 annotation [57]. Where alternate splice variants were present between samples as determined from RNAseq analysis (see below) only genes primarily sharing the same model were utilised to ensure accurate boundary comparisons i.e. Transcription Start Sites.

Continuous size variation plots (2D and 3D) were produced using a bespoke package (BAM2SizePlot.py) producing visualisations utilising paired-end insert distance to determine particle size and can be obtained at https://github.com/ChromatinCardiff/ALD.

RNAseq mapping was performed with Tophat2 [58], resulting in 78.40% alignment to the TAIR10 reference Colombia-0 genome [54] and the ARAPORT11 annotation [57]. Determination of isoform presence was achieved with cufflinks [59] and only primary isoforms shared between all samples were utilised as gene feature boundaries to ensure error was not induced between splice variants (n = 21,314). Quantitative assessment was performed with HTseq [60] and analysed with edgeR [61,62].

## Supporting information

S1 Fig Table: Nucleosomal and subNSP abundance throughout the Arabidopsis thaliana genome. (XLSX)

S2 Fig Mono-nucleosomal coverage at intron-exon boundary. (TIFF)

S3 Fig Boxplot demonstrating differential distance of +1 nucleosome from TSS between Light and dark grown samples in correlation to change in expression. (TIFF)

S4 Fig Athamap motif matrices predicting TFBS for PIF3 [42], PIF4 [44] and CCA1 [45]. (TIFF)

S5 Fig TF encoding genes with higher expression in dark grown samples did not demonstrate differential patterning at TF binding sites. (TIFF)

S6 Fig Table: MNase-seq and RNAseq read counts and mapping success. (XLSX)

S7 Fig Total Coverage (TC) quantification method and gene example. (TIFF)
