# Supplementary figures and images for: Genome-wide chromatin mapping with size resolution reveals a dynamic sub-nucleosomal landscape in Arabidopsis
Source: PLoS Genet. 2017 Sep 13;13(9):e1006988. doi: 10.1371/journal.pgen.1006988 (PMC5597176; doi:10.1371/journal.pgen.1006988)

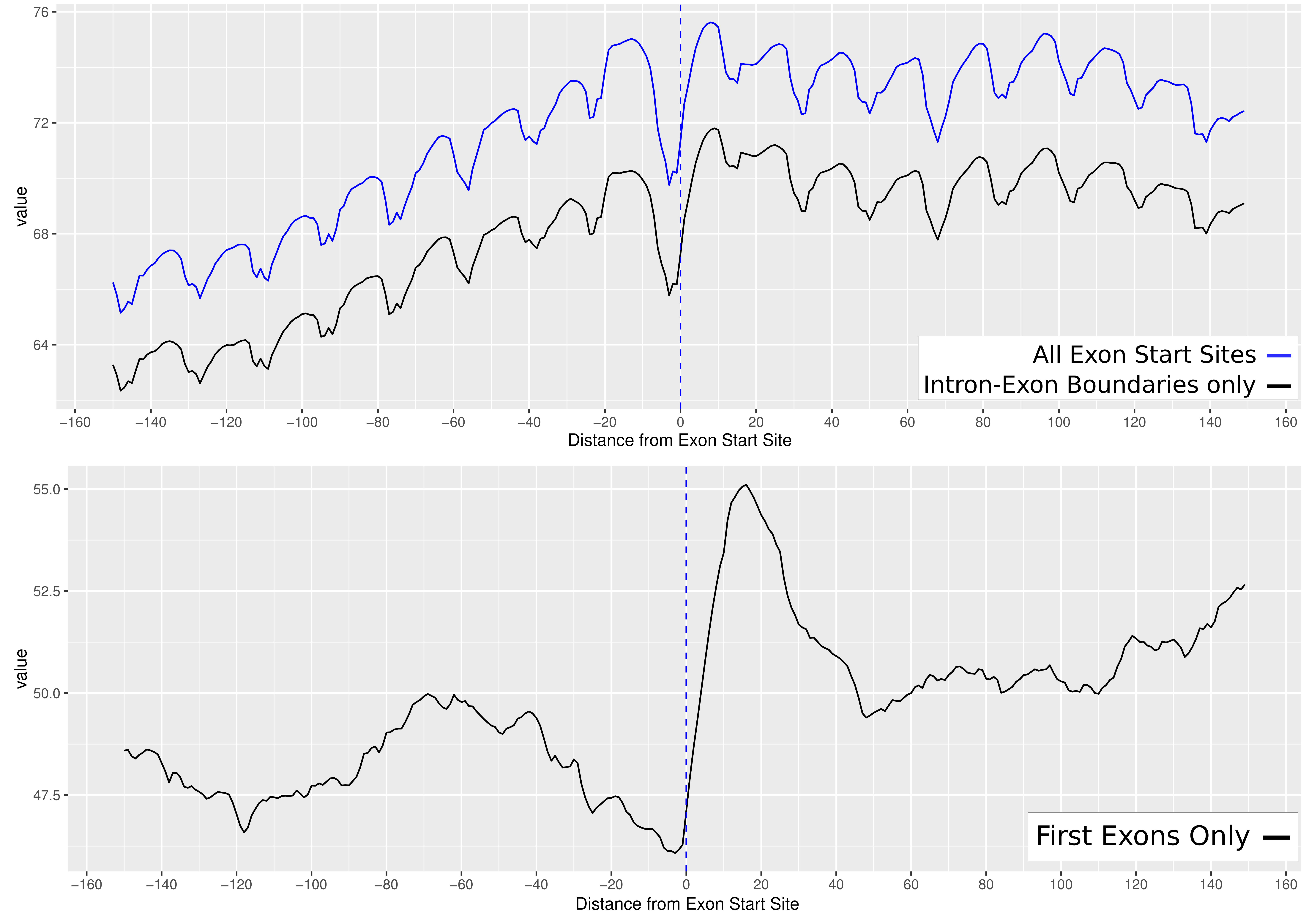

Supplement: S2 Fig — (TIFF) [file pgen.1006988.s002.tiff]

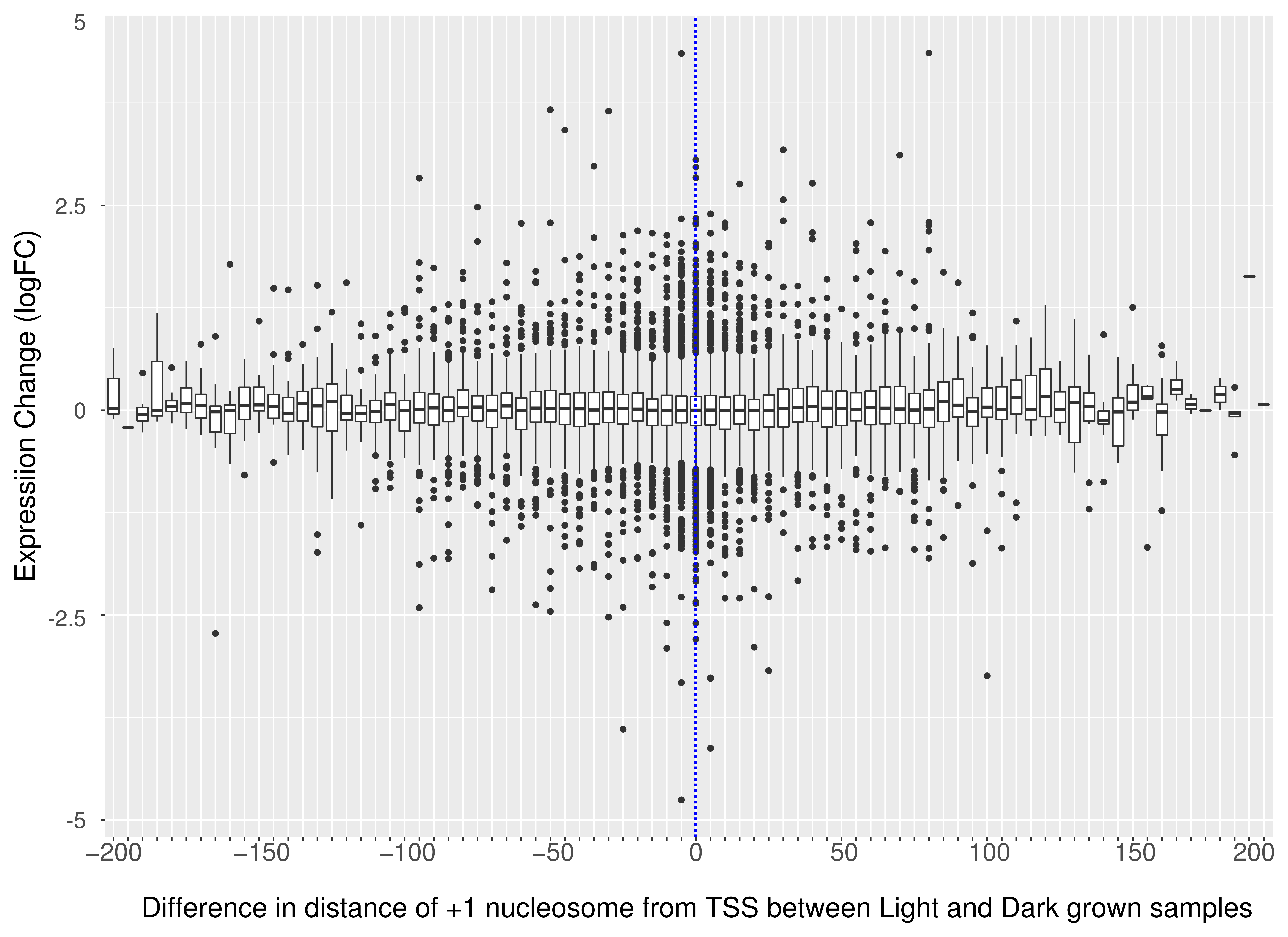

Supplement: S3 Fig — (TIFF) [file pgen.1006988.s003.tiff]

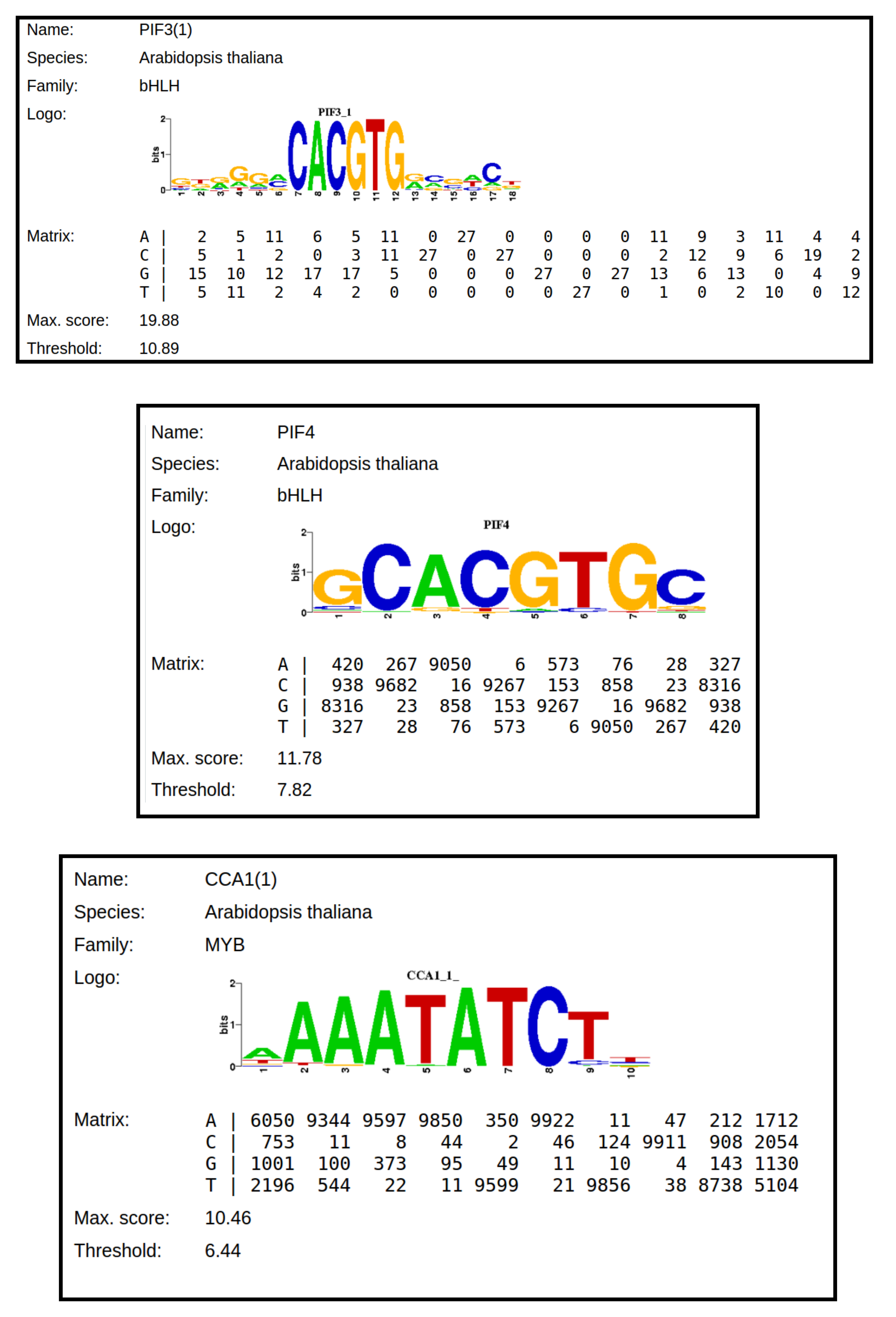

Supplement: S4 Fig — (TIFF) [file pgen.1006988.s004.tiff]

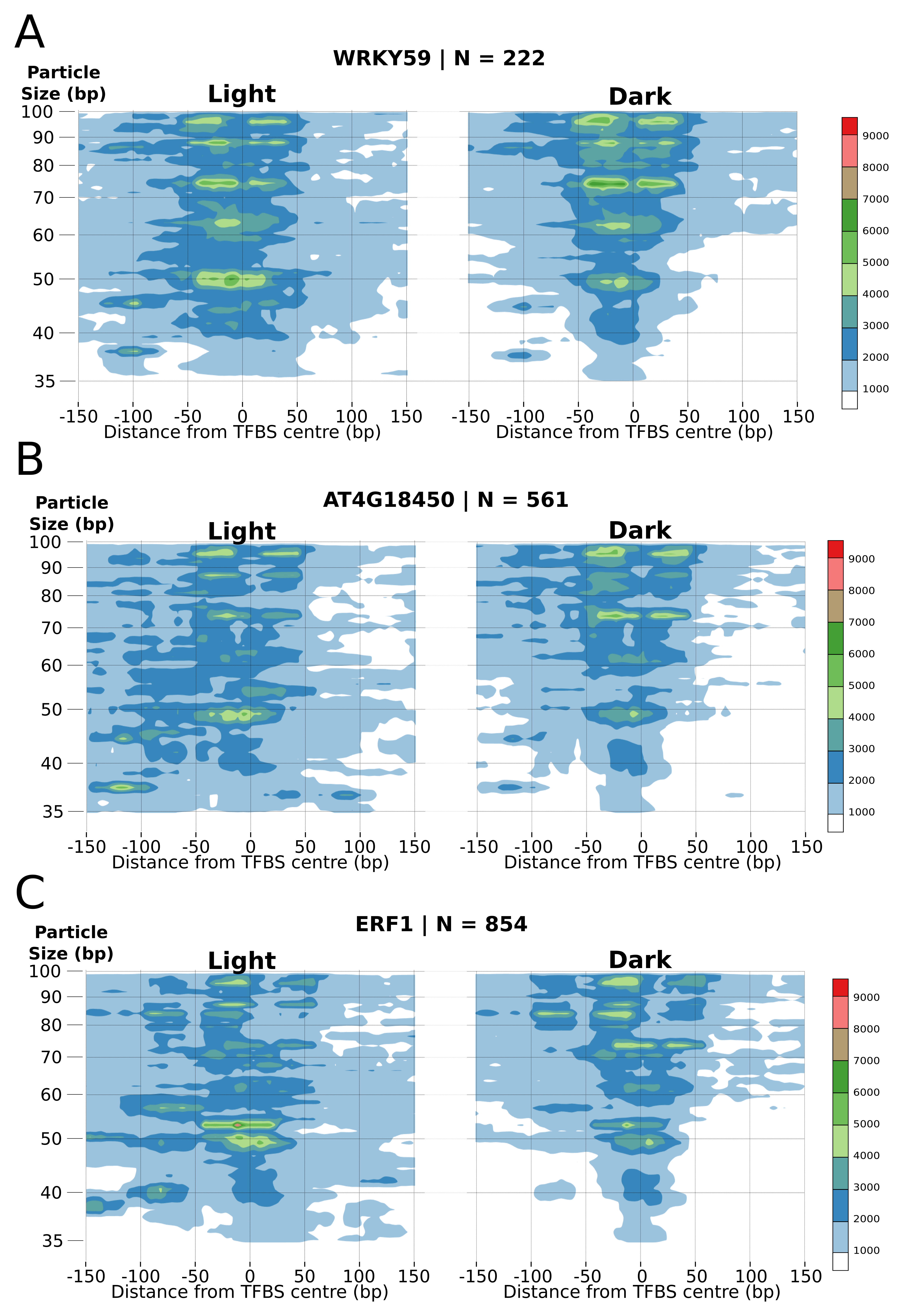

Supplement: S5 Fig — (TIFF) [file pgen.1006988.s005.tiff]

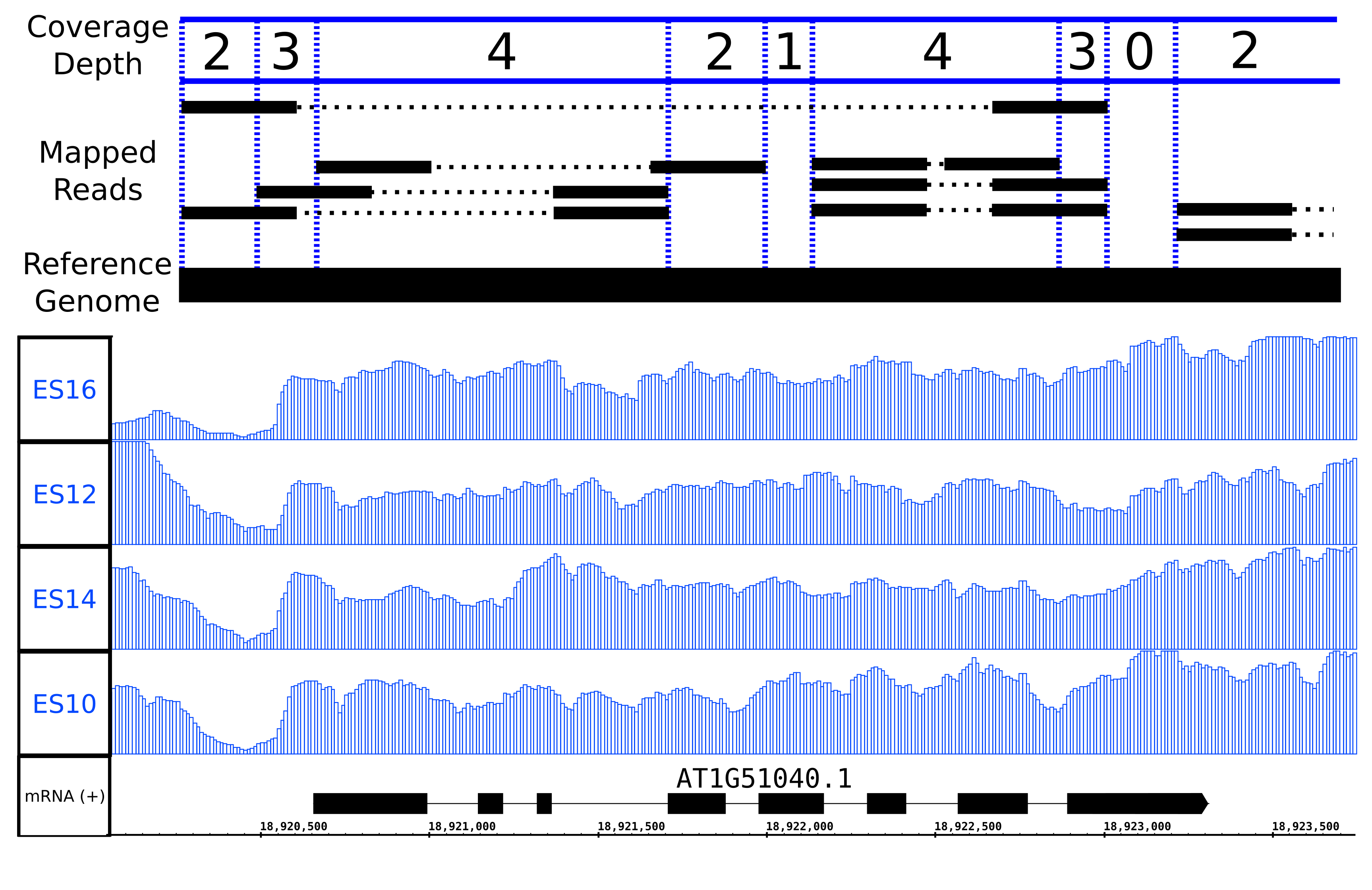

Supplement: S7 Fig — (TIFF) [file pgen.1006988.s007.tiff]
